# Large-scale screening of HIV-1 T-cell epitopes restricted by 12 prevalent HLA-A allotypes in Northeast Asia and universal detection of HIV-1-specific CD8^+^ T cells

**DOI:** 10.3389/fmicb.2025.1529721

**Published:** 2025-02-11

**Authors:** Yan Ding, Jialai Yan, Ling Huang, Jinhong Yu, Yandan Wu, Chuanlai Shen, Anning Fang

**Affiliations:** ^1^Department of Clinical Laboratory, The Second Hospital of Nanjing, Affiliated to Nanjing University of Chinese Medicine, Nanjing, Jiangsu, China; ^2^School of Medical Technology, Anhui Medical College, Hefei, Anhui, China; ^3^Department of Microbiology and Immunology, Medical School of Southeast University, Nanjing, Jiangsu, China; ^4^School of Basic Medicine, Anhui Medical College, Hefei, Anhui, China

**Keywords:** HIV-1, T-cell epitope, HLA-A, antigen-specific T-cell detection, CD8^+^ T-cell

## Abstract

**Background:**

Although the immune response of host T cells to human immunodeficiency virus (HIV) significantly influences the progression of the infection, the development of T-cell-based vaccines and therapies, as well as the clinical evaluation of specific T-cell functions, is currently markedly hindered by the absence of broad-spectrum, functionally validated HIV T-cell epitopes that account for the polymorphisms of the human leukocyte antigen (HLA) within an indicated geographic population. This study aimed to identify T-cell epitopes derived from the GP160, GAG, and POL proteins of the HIV-1 strain, specifically linked to 12 prevalent HLA-A allotypes, that collectively represent approximately 91% of the total gene frequency in Northeast Asian populations.

**Methods:**

A total of 134 epitopes were predicted *in silico* and selected as potential candidates for further validation. Subsequently, peripheral blood mononuclear cells (PBMCs) were collected from 96 individuals with HIV-1 and cocultured *ex vivo* with each epitope candidate peptide, followed by the detection of activated CD8^+^ T cells. Peripheral blood mononuclear cells (PBMCs) were collected from 96 individuals with HIV-1 and cocultured *ex vivo* with each candidate peptide epitope, followed by the detection of activated CD8+ T cells. A total of 69 epitopes were validated as real-world HIV T-cell epitopes presented by 12 dominant HLA-A allotypes. Furthermore, the HLA-A cross-restriction for each epitope candidate was identified through peptide competitive binding assays using 12 transfected HMy2.CIR cell lines.

**Results:**

A total of 45 epitopes demonstrated high affinity, while 31 epitopes displayed intermediate affinity. A broad-spectrum CD8^+^ T-cell epitope library containing 141 validated epitope peptides was used to universally detect HIV-1-specific CD8^+^ T cells *via* peptide-PBMC *ex vivo* coculture and intracellular IFN-*γ* staining. In 52 people with HIV-1, the number of reactive HIV-1 specific CD8^+^ T cells was significantly higher in the CD4^+^ T-cell-high patient group compared to the CD4^+^ T-cell-low patient group, and it correlated with the CD4^+^ T-cell-low patient group (<200/μL).

**Conclusion:**

This study provides a broad-spectrum CD8^+^ T-cell epitope library aimed at developing a T-cell-directed HIV vaccine that offers high population coverage in Northeast Asia. In addition, it establishes a universal detection method for the clinical assessment of HIV-1-specific CD8^+^ T-cell responses.

## 1 Introduction

Human immunodeficiency virus (HIV) destroys the immune system by targeting CD4^+^ T cells, CD4^+^ monocytes, and B cells. The host can endure lifelong infections and may ultimately die from complications, including opportunistic infections, lymphomas, and hematologic malignancies (Ghosn et al., 2018). Due to the high variability and glycosylation of the HIV envelope protein and the persistence of the HIV reservoir, the development of effective vaccines to prevent or cure HIV infection is currently limited (Bailón et al., 2022; Frank et al., 2024; Migueles et al., 2023; Wang et al., 2023). Novel vaccines and therapeutics remain to be further developed.

HIV-specific CD8^+^ T cells can inhibit viral replication through the direct cytotoxic action against HIV-infected CD4^+^ cells and the secretion of soluble cytokines. Therefore, they play a crucial role in immunity against HIV infection and are vital indicators for evaluating clinical efficacy, prognosis of recurrence, and vaccine effectiveness (Collins et al., 2020; Murakoshi et al., 2019; Mutascio et al., 2023; Nguyen et al., 2022; Perdomo-Celis et al., 2022; Warren et al., 2019). However, there is still no universally effective method for detecting HIV-specific CD8^+^ T cells in random patients within a specific geographic region due to the polymorphism of human leukocyte antigens (HLAs) and the absence of broad-spectrum T-cell epitopes that can adequately represent the HLA genetic characteristics of the cohort. According to the HIV-1 Molecular Immunology Database,^1^ a total of 2048 CD8^+^ T-cell epitopes and 702 CD4^+^ T-cell epitopes of the HIV proteome have been reported; however, it is noteworthy that majority of these epitopes are predicted *in silico*. Only 239 CD8^+^ T-cell epitopes and 82 CD4^+^ T-cell epitopes have undergone validation through cell experiments, such as Elispot, HLA binding, or ICS methods. The majority of these CD8^+^ T-cell epitopes are presented by a few HLA molecules, such as HLA-B35, B5301, A6802, or B1502 (Chikata et al., 2023; Kuse et al., 2023; Nguyen et al., 2022; Valenzuela-Ponce et al., 2018). Therefore, these CD8^+^ T-cell epitopes are not suitable for the predominant HLA-A, -B, and -C allotypes of Chinese and Northeast Asian populations, where the predominant allotypes are HLA-A0201, A2402, A1101, B4601, B4001, and B1302.^2^ In contrast, the validated T-cell epitopes derived from the HIV-1 A, B, C, D, and CRF01_AE correspond to 11.43, 58.26, 21.69, 4.96 and 3.65%, respectively, as summarized based on the data available on the website (see text footnote 1). The majority of the studies have focused on the prevalent HIV-1 subtype B in Europe and the United States. On the contrary, there is a lack of studies on the prevalent subtypes in China, where CRF01_AE, CRF07_BC, CRF08_BC, and B account for 39.0, 35.6, 8.9, and 5.5% of the total patient population, respectively (Bugembe et al., 2020; Kaseke et al., 2021; Kinloch et al., 2019). Overall, the reported T-cell epitope profile of the HIV proteome does not cover the HLA genetic polymorphism or the dominant genotypes of HIV-1 in China and Northeast Asia; thus, it cannot be used to establish a universal HIV-specific CD8^+^ T-cell detection system suited for this demographic population.

This study aimed to screen HIV-1 CD8^+^ T-cell epitopes restricted by 12 prevalent HLA-A allotypes, which have a total gene frequency of approximately 91% in Chinese and Northeast Asian populations (see text footnote 2). A total of 69 epitopes were ultimately validated *via in silico* prediction, peptide cocultures with patient peripheral blood mononuclear cells (PBMCs), and peptide competitive binding assays with HLA molecules. Furthermore, a universal detection system for HIV-1-specific CD8^+^ T cells was first established using validated broad-spectrum T-cell epitopes and intracellular IFN-*γ* staining, followed by tests conducted in 52 people with HIV-1.

## 2 Methods

### 2.1 Patient cohort and ethical approval

Individuals with HIV-1 were recruited for this study. Fresh peripheral blood samples were collected between 2023 and 2024 from the Department of Clinical Laboratory of Nanjing Second Hospital. Individuals with HIV-1 exhibited clinical, biochemical, Western blot, and virological evidence of HIV infection. The exclusion criteria for the study included infection with hepatitis viruses, tuberculosis, autoimmune diseases, or malignant tumors. The study was conducted in accordance with the principles of the Declaration of Helsinki, and the collection and use of human blood samples were approved by the Clinical Ethics Committee of the Second Hospital of Nanjing (reference No.: 2023-LK-kt053). Since these blood samples were biological specimens collected from prior clinical tests, informed consent was waived for the patients; however, consent was obtained from the Clinical Ethics Committee of the Second Hospital of Nanjing.

### 2.2 *In silico* prediction of CD8^+^ T-cell epitopes and peptide synthesis

The sequences of the GP160, GAG, and POL proteins of HIV-1 were obtained from the reference strains CRF01_AE CM240 (U54771) and CRF07_BC 97CN54 (AF286226),^3^ which are the predominant HIV subtypes in Asian populations. The 9-mer and 10-mer T-cell epitopes from the three proteins, presented by different HLA-A allotypes, were predicted using four virtual prediction tools such as SYFPEITHI, IEDBANN, SMM, and EPIJEN. For each HLA-A allotype and protein, up to 20 peptides displaying the highest affinity in at least two prediction tools were selected as epitope candidates for further validation. The peptides were synthesized by China Peptides Co., Ltd. (QYaoBio, Suzhou, China) and demonstrated a purity exceeding 95% as confirmed by HPLC and mass spectrometry analysis.

### 2.3 Peptide-PBMC coculture experiment and HLA-A genotyping

Briefly, PBMCs were routinely prepared from people with HIV-1 blood samples *via* Ficoll-Paque density-gradient centrifugation and cocultured in 96-well U-bottom plates (4 × 10^5^ PBMCs/well) with the indicated T-cell epitope candidate peptide (20 μg/mL) for 15–20 h in the RPMI 1640 medium supplemented with 10% fetal bovine serum at 37°C and 5% CO_2_. In parallel, negative control wells (PBMCs alone) and positive control wells (2 × 10^5^ PBMCs/well with phytohemagglutinin (PHA), 10 μg/mL) were also established. A mixture of BFA/Monensin (1000x) (Cat 420701, Biolegend, USA) was subsequently added to each well for an additional 6-h coculture. Then, the cells were harvested, washed, blocked with human Fc receptor-blocking reagent (Miltenyi Biotec, Shanghai, China) for 20 min at 4°C, and stained with antibodies against human CD3 (FITC-labeled, BioLegend) and CD8 (APC-labeled, BioLegend) for 30 min at 4°C. After washing, the cells were fixed, permeabilized using a Fix&Perm kit (Multi Sciences, Hangzhou, China), and incubated with a PE-conjugated anti-human IFN-*γ* antibody (BD Bioscience) for another 30 min at 4°C. Subsequently, the cells were analyzed by flow cytometry to determine the frequencies of IFN-γ^+^ cells in the CD3^+^/CD8^+^ populations.

For each sample, genomic DNA was extracted from PBMCs and HLA-A allele typing was carried out using PCR-sequencing-based typing (PCR-SBT) with the primers recommended by the International HLA Working Group (IHWG). Sequence alignment was conducted using the SOAPTying software.

### 2.4 Peptide competitive binding with HLA-A molecules

In our previous studies, twelve Hmy2.CIR cell lines expressing the indicated HLA-A allotype were generated, and the reference peptides bound to each HLA-A allotype were also selected (Ding et al., 2022). Briefly, 25 μL of T-cell epitope candidate peptide (5 or 15 μM, unlabeled) and 25 μL of the corresponding FITC-labeled reference peptide (300 nM) were added to a 96-well U-bottom culture plate. Moreover, the indicated CIR cell line was subjected to washing with an acid buffer for a duration of 1 min, and the final concentration of cells was adjusted to 4 × 10^5^/mL using IMDM that contained recombinant human β2-m (1 μg/mL). The cells were then added to each well (4 × 10^4^/well) and cocultured with the reference peptide and T-cell epitope candidate peptide for 24 h at 4°C. The negative control (cells alone) and positive control (cells with FITC-labeled reference peptide) were set up in parallel. Finally, the plate was washed twice with 200 μL of ice-cold 0.5% BSA-PBS (1,000 rpm for 5 min per wash). Afterward, flow cytometry analysis was performed to calculate the competitive binding inhibition.

The IC_50_ is the concentration of an unlabeled T-cell epitope peptide required to inhibit the binding of a FITC-labeled reference peptide to the HLA-A molecule by 50%, and this value is derived from the competitive binding inhibition percentage (%) of the T-cell epitope peptide at 5 and 15 μM. As described in previous studies (Ding et al., 2022; Wu et al., 2024), the binding affinity of a T-cell epitope candidate peptide to an indicated HLA-A allotype relative to the reference peptide was assessed using IC_50_, and the results were categorized into four classes: high, intermediate, low, and no affinity.

### 2.5 Quantitative detection of HIV-specific CD8^+^ T cells *via* intracellular IFN-*γ* staining and flow cytometry

In total, 141 validated broad-spectrum CD8^+^ T-cell epitope peptides were used in the detection system and grouped into 3 peptide pools according to the derived protein (GP160, GAG, and POL). PBMCs were routinely prepared from blood samples of individuals with HIV-1. For each sample, PBMCs were seeded into 5 wells in 96-well U-bottom plates (4 × 10^5^ cells/100 μL/well), and cocultured with each peptide pool (2 μg/peptide/well), no peptide mixture (negative control, PBMCs alone), or PHA mixture (positive control, PBMCs with 2.5 μg/well PHA) for 15–20 h in serum-free RPMI 1640 medium in a 5% CO_2_ incubator at 37°C. DMSO was added to the negative control wells to match the DMSO concentration in the peptide pool/PBMC coculture wells. Then, a BFA/Monensin (1000x) (Cat 420701, Biolegend, USA) was added to each well for another 6-h culture. Subsequently, the cells were harvested, washed, and blocked with anti-human CD16/CD32 (Miltenyi Biotec, Shanghai, China) for 20 min, followed by staining with FITC-labeled anti-human CD3 (BioLegend) and APC-labeled anti-human CD8a (BioLegend) for 30 min at 4°C. After washing, the cells were fixed and permeabilized using a Fix&Perm kit (MULTI SCIENCES) following the manufacturer’s protocol and further stained with PE-labeled anti-human IFN-*γ* (Bioscience) for 30 min at 4°C. Finally, flow cytometry analysis was performed to calculate the frequency of IFN-γ^+^ cells in the CD3^+^/CD8^+^cell populations. The counts of GP160-, GAG- or POL-specific CD8^+^ T cells in PBMCs = the CD8^+^ T-cell count in PBMCs × the corresponding frequency of IFN-γ^+^ cells in CD3^+^ CD8^+^ populations in response to the GP160, GAG, or POL peptide pool. HIV-1-specific CD8^+^ T cells were the sum of GP160-, GAG-, and POL-specific CD8^+^ T cells in PBMCs.

### 2.6 Detection of clinical parameters

CD8^+^ T cells, CD4^+^ T cells, and white blood cells were quantified using the flow cytometry (FACS Calibur, BD Bioscience) detection system and SYSMEX XN-2000 hematology analysers (Sysmex, Kobe, Japan). The plasma HIV RNA virus load (pVL) was detected using Cobas AmpliPrep/ Cobas TaqMan v 2.0 instrument (Roche Molecular Systems, Branchburg, NJ, United States) and corresponding reagents. These real-time data from the clinical laboratory of Nanjing Second Hospital were collected at the time of testing for HIV-specific T cells in each patient.

### 2.7 Statistical analysis

Statistical analysis was performed using GraphPad Prism 9 (GraphPad, La Jolla, CA, USA). The data are presented as the means (±standard error of the mean, SEM). When two groups were analyzed, the *t*-test was used for normally distributed or approximately normally distributed data. Multivariate linear regression analysis was performed to analyze the HIV-specific CD8^+^ T cell counts with clinical parameters. All the statistical analyses were two-sided tests, with a significance level of *p* ≤ 0.05, indicating statistical significance.

## 3 Results

### 3.1 A total of 134 CD8^+^ T-cell epitopes were selected as epitope candidates by *in silico* prediction

Using computational algorithms from four T-cell epitope prediction tools and adhering to a series of epitope screening principles as previously described (Ding et al., 2022; Wu et al., 2024), 134 CD8^+^ T-cell epitopes presented by 12 dominant HLA-A allotypes were finally selected as epitope candidates of HIV-1 proteins after considering the length of each protein and the gene frequency of each HLA-A allotype. The 12 dominant HLA-A allotypes (A1101, A2402, A0201, A0207, A3303, A0206, A3001, A0203, A3101, A1102, A0101, and A0301, with a gene frequency of >1% for each allotype) covered approximately 91% of the Chinese cohort and Northeast Asian populations (see text footnote 2). The 134 epitope candidates include 22 that were cross-restricted by several HLA-A allotypes. Therefore, only 112 epitope candidates had to be synthesized as peptides for further validation, of which 37, 30, and 45 candidates were harbored by the GP160, GAG, and POL proteins, respectively (Supplementary Table S1). For predicting cross-reactivity, these 112 candidates were also assessed for their affinity with HLA-B and C allotypes by *in silico* prediction (Supplementary Table S2). The majority of 69 validated peptides are relatively higher conservation in 14 HIV-1 subtypes (Table 1 and Supplementary Figure S1).

**Table 1:** 69 CD8^+^ T-cell epitopes validated by peptide-PBMCs cocultures and peptide competitive binding assay.

Epitope name	Epitope sequence	HIV genotype	Predicted HLA restriction	HLA-A alleles of patients in peptide-PBMCs cocultures	Reported HLA restriction	Peptide competitive binding assay for HLA-A			
						High affinity	Inter affinity	Low affinity	No affinity
GP160 691-IV9-699 (P5)	IIFAVLSIV	AE/BC	A0201, A0203	A0206/2601, A0201/2401			A0203	A0201	
GP160 195-RV9-203 (P6)	RLINCNTSV^**C**^	AE	A0201, A0203, A0206	A0206/2601, A0201/2401	A02		A0203	A0206	A0201
GAG 29-YA9-37 (P7)	YMLKHLVWA	BC	A0201, A0203	A0201/2401					A0201>A0203
GAG 275-RL9-283 (P9)	RMYSPVSIL^**C**^	AE	A0201, A0203	A0201/0207, A0201/2402, A0201/2402, A0201/0301			A0203		A0201
GAG 77-SL9-85 (P10)	SLFNTVATL^**C**^^,^ ^**V**^	AE/BC	A0201, A0203, A0207	A0201/1101, A2402/0301	A02			A0203>A0201	A0207
POL 587-YV9-595 (P11)	YQLEKDPIV^**C**^	AE/BC	A0201, A0207	A0201/0207					A0201>A0207
POL 448-AT9-456 (P12)	ALTDIVPLT^**C**^	AE/BC	A0201	A0201/0207			A0201		
POL 336-YV9-344 (P15)	YQYMDDLYV^**C**^^,^ ^**V**^	AE/BC	A0201	A0201/6601	A02	A0201			
GP160 285-KK9-293 (P19)	KTIIVHLNK	AE	A1101	A3001/3002					A0201
GP160 669-IK9-677 (P20)	ITNWLWYIK^**C**^	AE/BC	A1101	A3001/3002		A1101			
GP160 221-CK10-230 (P23)	CTPAGYAILK^**C**^	AE/BC	A1101	A0201/2402		A1101			
GP160 247-SK9-255 (P25)	SVQCTHGIK^**C**^	AE	A1101	A1101/3101		A1101			
GP160 88-VK9-96 (P26)	VTENFNMWK^**C**^	AE/BC	A1101	A1101/3101	A11, A68	A1101			
GP160 36-TK10-45 (P27)	TVYYGVPVWK^**C**^^,^ ^**V**^	BC	A1101	A1101/3101	A03, A11, A6801	A1101			
GAG 477-EK9-485 (P29)	ELYPLTSLK	BC	A1101	A0207/2301, A1101/2402		A1101			
GAG 6-SK10-15 (P30)	SILRGGKLDK	BC	A1101	A0207/2301, A1101/2402				A1101	
GAG 473-TK9-481 (P31)	TSLPKQEQK	AE	A1101, A1102	A0207/2301, A1101/2402		A1101			A1102
GAG 384-IK9-392 (P32)	IVKCFNCGK^**C**^	BC	A1101	A0207/2301, A1101/2402, A1101/3101				A1101	
GAG 281-SK10-290 (P33)	SILDIRQGPK^**C**^	AE	A1101, A1102	A1101/3101, A1101/3101	A1101	A1101	A1102		
GAG 83-AR9-91 (P34)	ATLWCVHQR^**C**^	AE	A1101, A3303, A1102	A1101/3101	A1101	A3303	A1101		A1102
POL 395-TK10-404 (P35)	TVQPIQLPEK^**C**^	BC	A1101	A1101/3101		A1101			
POL 536-AK10-545 (P36)	ATESIVIWGK^**C**^	AE	A1101	A1101/3101, A1101/3101		A1101			
POL 318-AK9-326 (P40)	AIFQSSMTK^**C**^^,^ ^**V**^	AE/BC	A1101, A3001, A0301, A1102		A0301, A11, A3, A33, A6801	A1101>A3001	A0301		A1102
POL 899-AR9-907 (P41)	AVFIHNFKR^**C**^	AE/BC	A1101, A3303, A1102		A24,A11, A3	A1101>A3303			A1102
POL 576-FK9-584 (P44)	FVNTPPLVK^**C**^	AE/BC	A1101	A0201/3301	A11	A1101		A1102	
POL 675-QK9-683 (P45)	QIIEQLIKK^**C**^	BC	A1101	A1101/3101	A02, A3, A11	A1101			A1102
GP160 383-FF9-391 (P49)	FYCNTTKLF^**C**^	AE	A2402	A0201/1101			A2402		
GP160 581-RL9-589 (P50)	RYLKDQQLL^**C**^^,^ ^**V**^	BC	A2402	A0207/2601	A2301, A2402, A02		A2402		
GP160 674-WI9-682 (P51)	WYIKIFIMI^**C**^	AE	A2402	A0207/2601	A2402				A2402
GAG 273-MI10-282 (P52)	MYSPTSILDI^**C**^	BC	A2402	A0207/2601			A2402		
GAG 276-MI10-285 (P53)	MYSPVSILDI^**C**^	AE	A2402	A0207/2601		A2402			
GAG 28-KW9-36 (P54)	KYRLKHLVW	AE	A2402	A0201/1101			A2402		
GAG 28-HW9-36 (P55)	HYMLKHLVW	BC	A2402	A0201/2401, A0301/3303	A2301, A2402			A2402	
GAG 258-IL10-267 (P56)	IYKRWIILGL^**C**^^,^ ^**V**^	AE/BC	A2402	A0301/3303	A2402			A2402	
POL 498-TF9-506 (P58)	TYQIYQEPF^**C**^	AE/BC	A2402	A3301		A2402			
POL 565-YW10-574 (P60)	YWQATWIPEW^**C**^	AE/BC	A2402	A3301		A2402			
POL 48-RF9-56 (P61)	RQGTISFNF	BC	A2402	A0201/2402, A0301/3303				A2402	
POL 537-IL10-546 (P62)	IWGKTPKFRL^**C**^	AE/BC	A2402	A0201/1101				A2402	
POL 215-PI9-223 (P63)	PYNTPVFAI^**C**^	AE	A2402	A0201/2402			A2402		
POL 286-KI10-295 (P65)	KYTAFTIPSI^**C**^	AE	A2402	A1101/3101, A0301/3303	A2	A2402			
GAG 293-FY9-301 (P66)	FRDYVDRFY^**C**^^,^ ^**V**^	AE	A0101	A1101/3301, A3001	A0101, A02				A0101
GP160 187-YY9-195 (P68)	YSENSSEYY	BC	A0101	A3001, A2402/0201	A01	A0101			
POL 651-VI10-660 (P69)	VTDSQYALGI^**C**^	AE/BC	A0101	A0201/2402, A3001	A01, A24		A0101		
GP160 22-GV9-30 (P70)	GMLMISSAV	BC	A0203	A0201/2402		A0203			
GAG 272-KV9-280 (P71)	KIVRMYSPV^**C**^	AE	A0206	A0201/1101					A0206
POL 791-KV9-799 (P74)	KVILVAVHV^**C**^	AE	A0206	A3001/3101				A0206	
GP160 580-YL10-589 (P78)	YLKDQKFLGL^**C**^	AE	A0207	A0201/3301				A0207	
GP160 120-KL9-128 (P79)	KLTPLCVTL^**C**^	AE/BC	A0207	A0201/3301, A0201/3001	A02, A11			A0207	
POL 527-QV9-535 (P80)	QLTEVVQKV^**C**^	AE	A0207	A0201/3001				A0207	
POL 268-VF9-276 (P81)	VLDVGDAYF^**C**^	AE	A0207	A0201/2402	A02				A0207
GAG 18-KK9-26 (P83)	KIRLRPGGK^**C**^^,^ ^**V**^	AE/BC	A0301	A2401/3101	A30, A11, A0301, A3				A0301
GP160 801-LK9-809 (P84)	LLYWGQELK	AE	A0301	A2401/3101			A0301		
POL 710-KK9-718 (P85)	KLVSSGIRK^**C**^	AE/BC	A0301	A2401/3101				A0301	
GP160 342-KK9-350 (P87)	KVLKQVTEK	AE	A1102	A2401/3101					A1102
GAG 37-AA9-45 (90)	ASRELERFA^**C**^	AE/BC	A3001	A3301/3101			A3001		
GP160 701-RL9-709 (P91)	RVRQGYSPL^**C**^	AE/BC	A3001	A1101/3301, A0201/3001		A3001			
GP160 766-RA9-774 (P92)	RLRGFILVA	BC	A3001	A0207/1101				A3001	
POL 978-KK9-986 (P93)	KVVPRRKAK^**C**^^,^ ^**V**^	AE/BC	A3001	A1101/3301, A1101/3301	A3			A3001	
POL 690-KA9-698 (P94)	KVYLSWVPA^**C**^	AE	A3001	A0201/3001				A3001	
POL 823-KK9-831 (P95)	KLAGRWPVK^**C**^	AE/BC	A3001, A0301	A0201/3001, A0207/1101	A0301	A3001	A0301		
GAG 375-RR9-383 (P97)	RSNFKGSKR	BC	A3101			A3101			
POL 12-KR9-20 (P100)	KAREFSSER	BC	A3101	A0201/3101			A3101		
POL 324-MR9-332 (P102)	MTKILEPFR^**C**^^,^ ^**V**^	AE/BC	A3101, A3303	A1101/3101	A0301	A3303>A3101			
GAG 139-MR9-147 (P103)	MVHQPISPR^**C**^	BC	A3101, A3303, 1102	A1101/3101, A0201/3101		A3303>A3101			A1102
GAG 14-DR9-22 (P104)	DAWEKIRLR^**C**^	AE	A3303	A0201/2402, A0201/0207, A0201/2402		A3303			
GAG 366-QR10-375 (P105)	QTNSAILMQR	BC	A3303	A0201/0207, A0201/2402				A3303	
GP160 428-MR9-436 (P106)	MYAPPISGR	AE	A3303	A0201/2402, A0201/2402		A3303			
POL 893-MR10-902 (P109)	MAVFIHNFKR^**C**^	BC	A3303	A2402/1101, A1101/3303		A3303			
GAG 142-MR9-150 (P111)	MAHQPLSPR^**C**^	AE	A3303, A1102	A1101/3301		A3303			A1102

Reported: Previously reported epitopes restricted by HLA-A allotypes.

Some peptides have been reported that we enrich and redefine their HLA-A restriction.

AE, CRF01_AE; BC, and CRF07_BC.

C Relatively conserved sequences according to homologous analysis in 14 HIV-1 genotypes.

V Epitopes that are included in the relevance of existing vaccines.

### 3.2 In total, 69 epitope candidates were validated as immunogenic epitopes *via* peptide-PBMC *ex vivo* coculture experiments and IFN-*γ* intracellular staining

**Figure 1:**
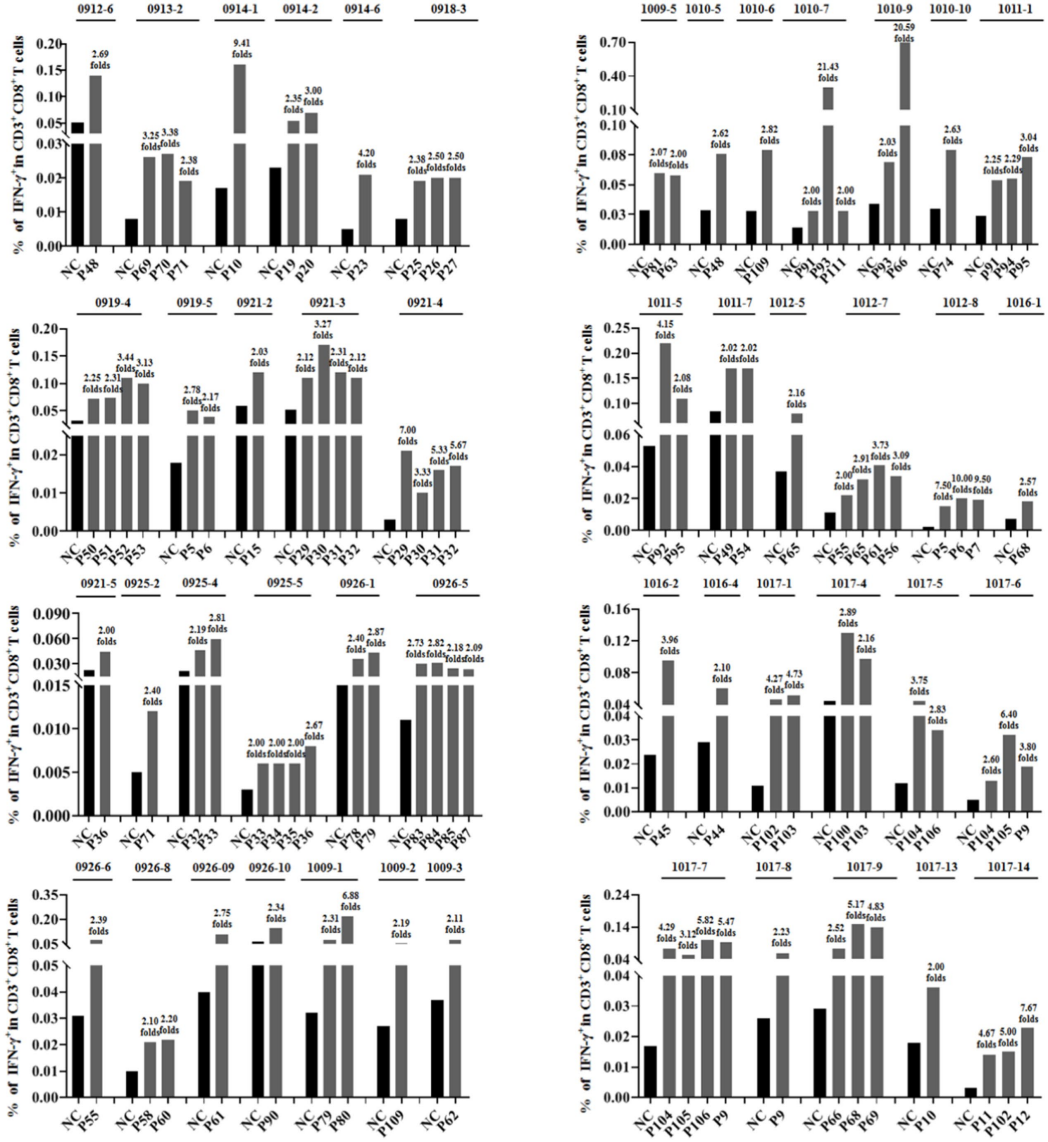
Sixty-nine epitope candidates were validated as real-world T-cell epitopes by peptide-PBMC *ex vivo* coculture experiments and intracellular IFN-*γ* staining. The PBMCs from 96 people with HIV-1 were cocultured for 20 h with the corresponding epitope candidate peptides, which could be represented by the patient’s HLA-A allotypes as virtually predicted. A total of 46 PBMCs from the peptide-PBMC cocultures presented positive T-cell responses. The frequencies of IFN-γ^+^ cells in the CD3^+^/CD8^+^ populations of each validated epitope and its negative control well in each PBMC sample are presented. NC, negative control well without peptide.

To further verify whether the epitope candidates could elicit memory CD8^+^ T-cell activation, PBMCs from 96 individuals with HIV-1 were collected and co-incubated *ex vivo* with the corresponding epitope candidate peptides, which were virtually predicted based on the patient’s HLA-A allotypes. The frequency of IFN-*γ*^+^ cells in the CD3^+^/CD8^+^ populations was assessed using intracellular IFN-*γ* staining and flow cytometry. When the frequency of IFN-γ^+^/CD8^+^ T cells in the coculture well was 2-fold greater than that in the negative control well, which consisted solely of PBMCs, the epitope candidate peptide present in the coculture was designated as the immunogenic epitope. This finding suggests the existence of the peptide-specific memory CD8^+^ T-cell clones in the patient’s peripheral blood (Ding et al., 2022; Wu et al., 2024). In total, 46 patients’ PBMCs displayed positive CD8^+^ T-cell responses, 69 epitope candidates were defined as real-world immunogenic epitopes, and each epitope peptide stimulated CD8^+^ T-cell responses in one, two, or three patient samples (Table 1). A total of 44 out of 69 validated peptides have not been reported and 26 had been reported. Notably, 10 out of 26 reported peptides had been included in the relevance of existing vaccines. The frequencies of IFN-γ^+^ cells in the CD3^+^/CD8^+^ populations for each validated epitope and its negative control well in each peptide-PBMC coculture are presented in Figure 1. The flow cytometric dot plots of intracellular IFN-γ staining are presented in Supplementary Figure S2. The clinical data and HLA-A alleles of 46 patients whose PBMCs displayed positive CD8^+^ T-cell responses in peptide-PBMCs cocultures are shown in Supplementary Table S3.

### 3.3 Binding affinity and cross-binding of 112 epitope candidates with 12 HLA-A allotypes were analyzed using a peptide competitive binding assay

**Figure 2:**
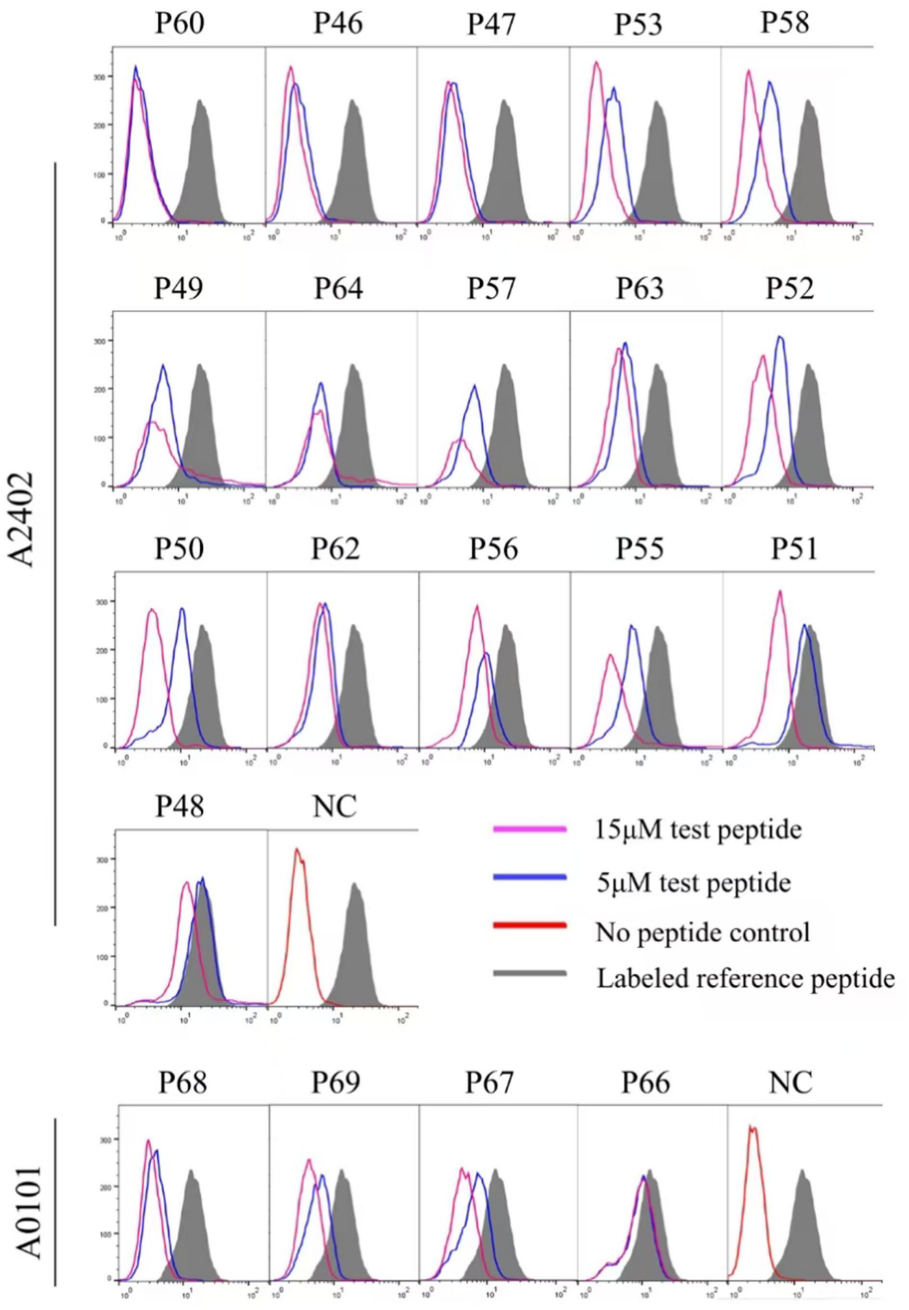
Binding affinity analysis of 112 HIV epitope candidates to the corresponding HLA-A molecules using competitive peptide binding assay. A series of unlabeled peptides and a fluorescent reference peptide were co-incubated with HMy2.CIR cell lines expressing the indicated HLA-A molecules. The competition binding inhibition (%) of each standby peptide at 5 and 15 μM was then calculated by determining the percentage of fluorescence-positive HMy2.CIR. The blue and violet lines are peak plots of cellular fluorescence intensity at 5 and 15 μM of the peptide to be detected, respectively. The gray filled line represents the maximum cellular fluorescence (cells using only the fluorescent reference peptide); the red line in the NC plot represents the background fluorescence of cells using only the RPMI 1640 culture medium as a fluorescent negative control.

Twelve transfected HMy2.CIR cell lines, which exhibit constitutive expression of the indicated HLA-A allotype, were employed to evaluate the affinity between epitope candidates and their corresponding HLA-A molecules through a peptide competitive binding assay. The line diagrams from the flow cytometric analysis revealed that, compared with the reference peptide, majority of the competing peptides led to diminished fluorescence in the indicated CIR cell lines (leftward shift of the fluorescence peak) (Figure 2 and Supplementary Figure S2). This finding indicated that the candidate peptides could efficiently compete with the labeled reference peptide to bind the relevant HLA-A molecules, revealing their binding power with the indicated HLA-A molecules. Among the 112 epitope candidates, 45 high-affinity, 31 intermediate-affinity, 34 low-affinity, and 34 no-affinity candidates have defined in the competitive peptide binding assays, and the majority of the high-affinity or interaffinity epitope candidates contained conserved amino acids at the P2 position and COOH terminus (Table 2). Among the 69 epitopes validated in the peptide-PBMC coculture experiments, 44 showed high affinity or intermediate affinity for the relevant HLA-A allotypes (Table 1).

**Table 2:** Binding affinity of 112 HIV T-cell epitope candidates with 12 prevalent HLA-A allotypes.

HLA-A	Peptides	Affinity	5-μM inhibition (%)	15-μM inhibition (%)	Sequence
A0201	P16	High	65.79%	83.00%	Y**T**AFTIPS**I**
	P15	High	56.45%	83.00%	Y**Q**YMDDLY**V**
	P1	Inter	38.03%	78.46%	S**L**VDTIAIA**V**
	P12	Inter	28.02%	72.19%	A**L**TDIVPL**T**
	P14	Inter	27.62%	81.53%	Q**L**PEKDSWT**V**
	P3	Inter	26.15%	66.19%	S**L**AEGEII**I**
	P2	Inter	23.22%	70.46%	A**L**FYKLDI**V**
	P5	Low	20.55%	42.83%	IIFAVLSIV
	P18	Low	15.88%	41.90%	ALQDSGSEV
	P10	Low	15.75%	31.36%	SLFNTVATL
	P4	Low	10.81%	44.70%	QMQEDVISL
	P13	Low	10.68%	40.43%	TLWQRPLVTV
	P7	No	8.94%	16.50%	YMLKHLVWA
	P17	No	6.14%	15.08%	LLWKGEGAV
	P11	No	5.87%	28.56%	YQLEKDPIV
	P6	No	0.13%	23.62%	RLINCNTSV
	P8	No	0.00%	14.68%	ALQTGTEEL
	P9	No	0.00%	4.54%	RMYSPVSIL
	P19	No	0.00%	4.54%	KTIIVHLNK
A1101	P27	High	93.13%	94.17%	T**V**YYGVPVW**K**
	P42	High	89.88%	93.78%	S**V**PLDESFR**K**
	P35	High	88.71%	93.91%	T**V**QPIQLPE**K**
	P36	High	88.32%	90.66%	A**T**ESIVIWG**K**
	P26	High	86.63%	92.87%	V**T**ENFNMW**K**
	P44	High	84.55%	92.74%	F**V**NTPPLV**K**
	P41	High	83.77%	86.76%	A**V**FIHNFK**R**
	P33	High	83.25%	93.13%	S**I**LDIRQGP**K**
	P43	High	80.25%	90.14%	I**V**IWGKTP**K**
	P28	High	78.43%	92.22%	T**T**LFCASDA**K**
	P31	High	75.18%	86.50%	T**S**LPKQEQ**K**
	P37	High	74.03%	91.02%	G**S**NFTSAAV**K**
	P23	High	70.11%	88.06%	C**T**PAGYAIL**K**
	P20	High	69.85%	71.02%	I**T**NWLWYI**K**
	P40	High	67.90%	89.49%	A**I**FQSSMT**K**
	P45	High	62.96%	85.33%	Q**I**IEQLIK**K**
	P25	High	61.39%	85.59%	S**V**QCTHGI**K**
	P39	High	60.35%	86.24%	R**T**AHTNDV**K**
	P29	High	51.12%	79.86%	E**L**YPLTSL**K**
	P34	Inter	43.96%	77.78%	A**T**LWCVHQ**R**
	P22	Inter	43.18%	87.41%	G**T**YTPNGT**K**
	P21	Inter	29.66%	68.94%	S**V**IKQACP**K**
	P24	Low	20.55%	36.55%	SSWSNKSQK
	P32	Low	20.16%	36.81%	IVKCFNCGK
	P30	Low	19.77%	47.09%	SILRGGKLDK
	P38	Low	12.62%	33.56%	ASCDQCQLK
A2402	P60	High	97.52%	97.79%	Y**W**QATWIPE**W**
	P46	High	95.05%	99.52%	F**Y**CNTSGL**F**
	P65	High	93.12%	97.79%	K**Y**TAFTIPS**I**
	P47	High	87.46%	94.49%	N**Y**TNQIYE**I**
	P53	High	75.53%	98.09%	M**Y**SPVSILD**I**
	P58	High	54.14%	95.35%	T**Y**QIYQEP**F**
	P49	Inter	49.24%	60.49%	F**Y**CNTTKL**F**
	P54	Inter	35.82%	79.55%	K**Y**RLKHLV**W**
	P64	Inter	31.19%	50.22%	V**Q**MAVFIHN**F**
	P57	Inter	29.19%	70.74%	W**W**MEYWQAT**W**
	P63	Inter	25.74%	55.59%	P**Y**NTPVFA**I**
	P52	Inter	21.61%	79.43%	M**Y**SPTSILD**I**
	P50	Inter	15.60%	87.90%	R**Y**LKDQQL**L**
	P62	Low	21.17%	37.66%	IWGKTPKFRL
	P56	Low	20.61%	25.51%	IYKRWIILGL
	P55	Low	13.48%	31.20%	HYMLKHLVW
	P61	Low	12.82%	41.47%	RQGTISFNF
	P51	No	3.34%	29.97%	WYIKIFIMI
	P48	No	1.56%	6.91%	W**Y**IKIFII**I**
A0101	P68	High	83.75%	94.16%	Y**S**ENSSEY**Y**
	P69	Inter	35.65%	71.19%	V**T**DSQYALG**I**
	P67	Inter	21.42%	61.98%	E**A**DGKVSQN**Y**
	P66	No	5.38%	5.38%	FRDYVDRFY
A0203	P70	High	71.41%	85.50%	G**M**LMISSA**V**
	P6	Inter	15.81%	70.67%	R**L**INCNTS**V**
	P5	Inter	31.01%	64.62%	I**I**FAVLSI**V**
	P2	Inter	26.07%	61.90%	A**L**FYKLDI**V**
	P3	Inter	0.12%	51.10%	S**L**AEGEII**I**
	P9	Inter	0.00%	50.10%	R**M**YSPVSI**L**
	P10	Low	20.39%	42.50%	SLFNTVATL
	P1	Low	28.17%	31.88%	SLVDTIAIAV
	P7	No	3.83%	19.15%	YMLKHLVWA
	P8	No	0.00%	5.56%	ALQTGTEEL
	P4	No	0.00%	0.00%	QMQEDVISL
A0206	P72	Inter	40.91%	67.34%	W**I**ILGLNK**I**
	P73	Low	25.71%	27.84%	QIINMWQEV
	P6	Low	14.63%	41.77%	RLINCNTSV
	P74	Low	20.64%	13.64%	KVILVAVHV
	P71	No	6.54%	14.21%	KIVRMYSPV
	P75	No	3.69%	5.11%	KIILVAVHV
A0207	P79	Low	30.12%	33.92%	KLTPLCVTL
	P15	Low	20.10%	27.60%	YQYMDDLYV
	P80	Low	20.67%	25.60%	QLTEVVQKV
	P82	Low	13.67%	30.79%	YMEAEVIPA
	P76	Low	11.90%	30.60%	YVDRFFKTL
	P78	Low	11.50%	30.40%	YLKDQKFLGL
	P77	No	9.36%	23.40%	LVDTIAIAV
	P11	No	5.57%	12.66%	YQLEKDPIV
	P81	No	0.00%	23.16%	VLDVGDAYF
	P10	No	0.00%	7.34%	SLFNTVATL
	P14	No	0.00%	8.35%	QLPEKDSWTV
A0301	P95	Inter	28.94%	53.68%	K**L**AGRWPV**K**
	P84	Inter	25.67%	56.24%	L**L**YWGQEL**K**
	P40	Inter	22.40%	50.64%	A**I**FQSSMT**K**
	P85	Low	19.14%	38.04%	KLVSSGIRK
	P83	No	1.10%	24.97%	KIRLRPGGK
A1102	P33	Inter	16.76%	50.61%	S**I**LDIRQGP**K**
	P44	No	6.01%	26.12%	FVNTPPLVK
	P87	No	5.66%	16.76%	KVLKQVTEK
	P111	No	4.16%	20.57%	MAHQPLSPR
	P31	No	4.16%	18.03%	TSLPKQEQK
	P103	No	3.81%	13.40%	MVHQPISPR
	P34	No	3.12%	13.75%	ATLWCVHQR
	P40	No	2.89%	16.06%	AIFQSSMTK
	P41	No	1.73%	21.49%	AVFIHNFKR
	P86	No	1.04%	3.12%	AAGTGSSSK
	P45	No	0.12%	12.13%	QIIEQLIKK
	P88	No	0.00%	0.35%	RANSPTSRK
A3001	P91	High	92.43%	99.99%	R**V**RQGYSP**L**
	P95	High	83.72%	95.89%	K**L**AGRWPV**K**
	P40	High	60.44%	67.69%	A**I**FQSSMT**K**
	P90	Inter	17.73%	58.02%	A**S**RELERF**A**
	P94	Low	27.40%	45.93%	KVYLSWVPA
	P89	Low	27.40%	39.48%	RLRPGGRKK
	P92	Low	18.53%	39.48%	RLRGFILVA
	P93	Low	16.92%	41.10%	KVVPRRKAK
A3101	P97	High	66.61%	92.94%	R**S**NFKGSK**R**
	P103	High	65.42%	79.18%	M**V**HQPISP**R**
	P41	High	63.42%	96.01%	A**V**FIHNFK**R**
	P102	High	60.23%	88.35%	M**T**KILEPF**R**
	P98	Inter	47.87%	81.97%	R**A**KRRVVE**R**
	P100	Inter	42.88%	95.12%	K**A**REFSSE**R**
	P101	Low	32.90%	41.10%	NLKTGKYAR
	P112	Low	20.10%	25.70%	IQNFRVYYR
	P99	No	0.00%	15.00%	VTGIRKNYR
	P96	No	0.00%	0.00%	RIKCFNCGR
A3303	P110	High	78.19%	90.08%	T**F**YVDGAAS**R**
	P111	High	78.19%	89.21%	M**A**HQPLSP**R**
	P106	High	72.80%	81.52%	M**Y**APPISG**R**
	P104	High	71.78%	82.54%	D**A**WEKIRL**R**
	P41	High	71.01%	89.80%	A**V**FIHNFK**R**
	P103	High	70.24%	99.50%	M**V**HQPISP**R**
	P102	High	69.47%	69.98%	M**T**KILEPF**R**
	P34	High	63.83%	77.16%	A**T**LWCVHQ**R**
	P109	High	55.11%	56.12%	M**A**VFIHNFK**R**
	P112	Inter	48.71%	55.10%	I**Q**NFRVYY**R**
	P108	Low	35.63%	40.50%	QTVYALFYR
	P107	Low	34.61%	49.22%	NITGILLTR
	P105	Low	29.22%	45.63%	QTNSAILMQR

The binding affinity of the target peptide with the indicated HLA-A allotypes was assessed by the IC_50,_ which is the concentration of unlabeled target peptide required to inhibit the binding of the labeled peptide by 50%. IC_50_ < 5 μM (5-μM inhibition>50%) indicates high binding affinity, 5 μM < IC_50_ < 15 μM (5-μM inhibition <50%, but 15-μM inhibition >50%) indicates intermediate binding affinity, 5 μM inhibition 20–50% or 15-μM inhibition 30–50% indicates low binding affinity; 5-μM inhibition <20% or 15-μM inhibition <30% indicates no binding affinity.

These high- or interaffinity peptides carry the motif in P_2_ and at the COOH terminus (bold).

The majority of epitope candidates showed high- or interaffinity cross-binding to several HLA-A molecules to broaden the population coverage (Table 2). For example, POL 318-AK9-326 (P40) bound to three HLA-A allotypes (A1101, A3001, and A0301) with high affinity, whereas POL 324-MR9-332 (P102), GAG 139-MR9-147 (P103), and POL 899-AR9-907 (P41) bound to two HLA-A allotypes with high affinity (A3303 and A3101 for P102; A3303 and A3101 for P103; and A1101 and A3303 for P41). Taken together with the predicted HLA allotypes, previously reported HLA allotypes, and the HLA alleles of the patients with positive CD8^+^ T-cell response in the peptide-PBMC cocultures, these data suggested that the majority of the 69 validated epitopes could be cross-presented by several HLA-A allotypes, as summarized in Table 1, indicating that over 91% of the population coverage of these patients occurred in China or Northeast Asia.

### 3.4 Clinical detection of HIV-1 specific CD8^+^ T cells using 141 validated epitope peptides

To monitor HIV-1 specific cellular immunity comprehensively in Chinese patients carrying different HLA-A alleles, a broad-spectrum CD8^+^ T-cell epitope library was designed, which contained 69 epitopes that were validated herein by peptide-PBMC *ex vivo* cocultures and another 72 epitopes that were reported by other researchers or predicted *in silico* but confirmed using a peptide competitive binding assay in this study (Supplementary Table S4). Furthermore, the majority of the 72 peptides originated from high affinity with HLA-A allotypes by *in silico* prediction, inter/high affinity in peptide competitive binding assay experiment, or reported peptides restricted by low frequency of HLA-A subtypes. Due to the relatively low frequency of HLA-A subtypes in the population, immune responses to peptides could not be detected in small samples; they may be detected in larger samples, so all of them are included in peptide pools. The final library covered over 91% of the Chinese and Northeast Asian populations, and each epitope could cross-bind to several of the 12 predominant HLA-A allotypes (Table 3).

**Table 3:** Protein distribution and HLA-A restriction of 141 HIV-1 CD8^+^ T-cell epitope peptides used in clinical tests.

HLA-A allotype	Allele frequency	HIV CD8^+^ T-cell epitope peptides			Total
		Gp160 (857aa)	Gag (503aa)	Pol (1008aa)	
A0201	13.00%	6	4	8	18
A1101	16.50%	10	6	11	27
A2402	15.10%	6	5	9	20
A0203	2.30%	3	3	2	8
A0206	5.60%	2	2	2	6
A0207	6.70%	3	2	4	9
A3303	11.18%	3	5	5	13
A3001	11.10%	2	2	4	8
A3101	3.07%	2	3	3	8
A0301	2.90%	2	2	3	7
A1102	0.30%	2	6	3	11
A0101	3.70%	2	2	2	6
Total	91.45%	43	42	56	141

**Figure 3:**
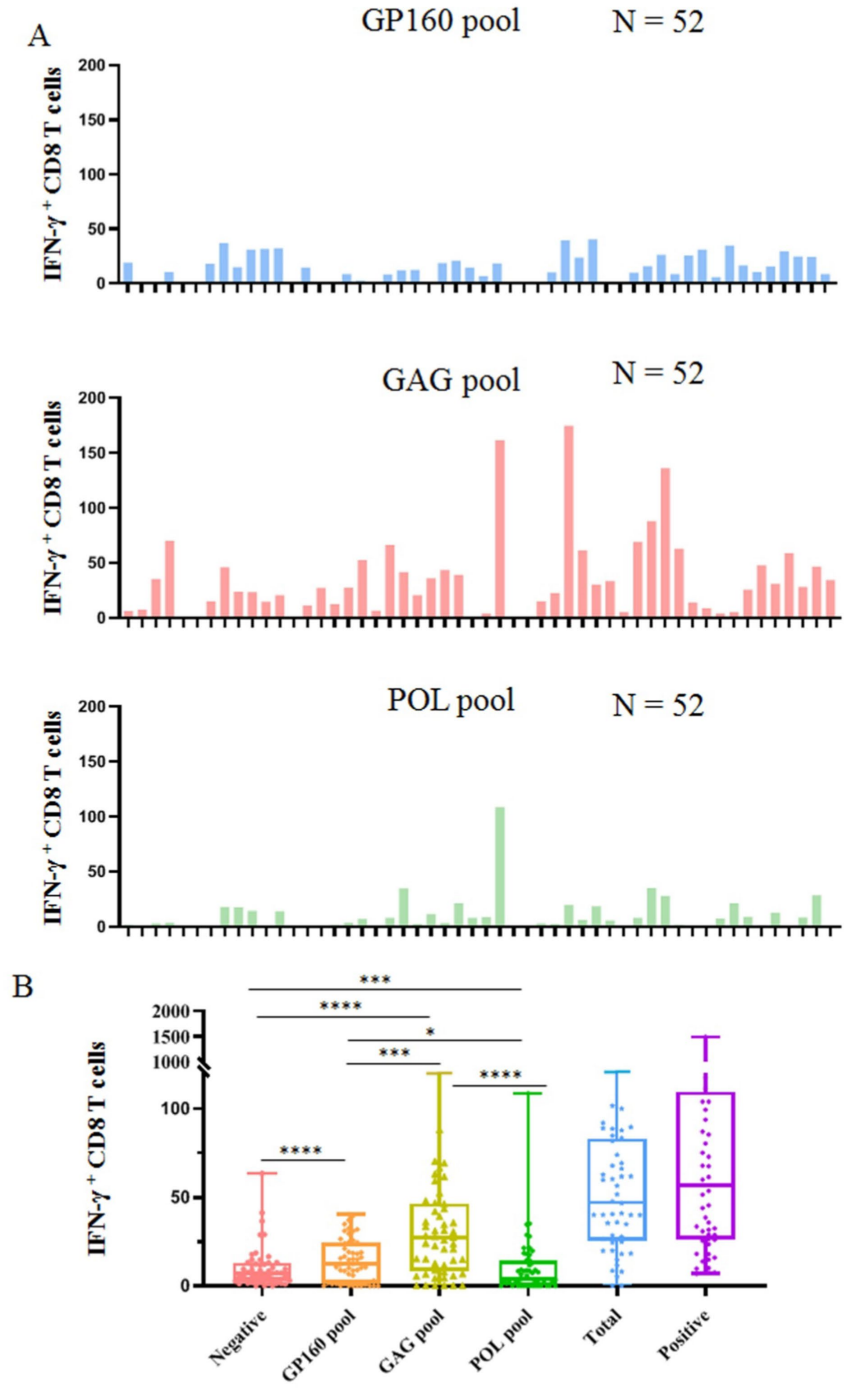
Clinical detection of HIV-1 specific CD8^+^ T cells using 141 validated epitope peptides. The epitope peptides were grouped into three pools according to the derived protein (GP160, GAG, and POL) and *ex vivo* co-incubated for 15–20 h with PBMCs from 52 people with HIV-1 followed by IFN-γ ICS. **(A)** The histogram plots show the count of INF-γ^+^ CD8 T cells (negative control values were excepted) in the PBMCs of 52 people with HIV-1 after stimulation with three peptide pools. **(B)** The comparison of the count of IFN-γ^+^ CD8 T cells in the negative control, three peptide pools, the sum of three peptide pools, and positive control.

The epitope peptides were then grouped into three pools according to the derived protein (GP160, GAG, and POL), and co-incubated *ex vivo* for 15–20 h with PBMCs from patients, followed by IFN-*γ* ICS to enumerate the memory HIV-1 specific CD8^+^ T cells in the peripheral blood. In total, 52 people with HIV-1 from another cohort were tested, and their clinical baseline features, HIV-1 specific CD8^+^ T-cell counts, and stratification analysis results are displayed in Figure 3 and Table 4. GAG-specific CD8^+^ T-cell counts were greater than GP160- or POL-specific CD8^+^ T-cell counts. The IFN-γ ICS dot plots of representative patients are shown in Supplementary Figure S3. The HIV-1-specific CD8^+^ T-cell and GP160-, GAG-, and POL-specific CD8^+^ T-cell counts were calculated as previously described. The patients were further grouped into a CD4^+^ T-cell-high group (≥200/μL) and a CD4^+^ T-cell-low patient group (<200/μL). As shown in Table 4, the number of HIV-1-specific CD8^+^ T cells was much greater in the CD4^+^ T-cell-high patient group than in the CD4^+^ T-cell-low patient group (*p* = 0.023), while the number of GP160- or GAG-special CD8^+^ T cells was also much greater in the CD4^+^ T-cell-high patient group than in the CD4^+^ T-cell-low patient group (*p* < 0.05).

**Table 4:** Baseline clinical data of the enrolled people with HIV-1 (mean ± SEM) and stratification analysis of HIV specific CD8^+^ T-cell reactivity.

Clinical features	CD4^+^ T cell-low group (<200/μL)	CD4^+^ T-cell-high group (≥200/μL)	*t*	*p*
Number (n)	17	35	/	/
Male/Female (n)	16/1	31/4	/	/
Age (years)	50.88 ± 4.22	44.83 ± 2.64	1.263	0.213
HIV RNA (log10 copies/mL)	2.95 ± 0.46	1.67 ± 0.18	3.101	**0.003**
CD4 counts (μL)	91.18 ± 15.22	498.9 ± 34.05	8.118	**<0.000**
CD8 counts (μL)	573.8 ± 87.13	939.6 ± 79.94	2.814	**0.007**
CD4/CD8 ratio	0.30 ± 0.11	0.72 ± 0.09	2.748	**0.008**
WBC counts (10^9^/L)	4.86 ± 0.55	6.31 ± 0.31	2.506	**0.016**
GP160-specific CD8^+^ T cells (10^4^/L)	10.12 ± 2.76	16.98 ± 1.70	2.208	**0.032**
GAG-specific CD8^+^ T cells (10^4^/L)	21.02 ± 4.26	42.26 ± 6.99	2.023	**0.048**
POL-specific CD8^+^ T cells (10^4^/L)	5.99 ± 1.80	12.53 ± 3.25	1.349	0.184
HIV-1 specific CD8^+^ T cells (10^4^/L)	37.00 ± 6.67	72.11 ± 9.86	2.349	**0.023**

The count of GP160-, GAG- or POL-specific CD8^+^ T cells in PBMCs = the CD8^+^ T-cell count in PBMCs × the corresponding frequency of IFN-γ^+^ cells in CD3^+^CD8^+^ populations in response to the GP160, GAG or POL peptide pool; the number of HIV-1-specific CD8^+^ T cells was the sum of the number of GP160-, GAG- and POL-specific CD8^+^ T cells in PBMCs. *P*<0.05 was highlighted in bold.

Moreover, multivariate linear regression analyses were performed, as shown in Table 5. The HIV-1-specific CD8^+^ T-cell count was used as the dependent variable, whereas HIV RNA load, CD4^+^ T-cell count, CD8^+^ T-cell count, CD4^+^/CD8^+^ ratio, WBC count and CD4^+^ T-cell-low patient group (< 200/μL) were used as independent variables. The results revealed that the regression model was accurate (*F* = 2.339, *p* = 0.048). The *p* values obtained for the HIV RNA load, CD4^+^ T-cell count, CD8^+^ T-cell count, CD4^+^/CD8^+^ T-cell ratio, WBC count and the incidence of CD4^+^ T-cell-low patient group (<200/μL) were 0.19, 0.14, 0.22, 0.68, 0.31, and 0.05, respectively, suggesting that HIV-1 specific CD8^+^ T-cell count was significantly correlated with the incidence of CD4^+^ T-cell-low patient group (<200/μL). When the CD4^+^ T-cell-low patient group (<200/μL) parameter was excluded, the multivariate linear regression analysis reasonably revealed a correlation between the HIV-1-specific CD8^+^ T-cell count and the CD8^+^ T-cell count (*F* = 5.027, *p* = 0.03). In another regression model in which the GAG-specific CD8^+^ T-cell count was used as the dependent variable, the GAG-specific CD8^+^ T-cell count was also significantly correlated with the CD8^+^ T-cell count (*F* = 5.76, *p* = 0.02).

**Table 5:** Multivariate linear regression model predicting HIV-1 specific CD8^+^ T cells correlation to clinical data among people with HIV-1, *n* = 52.

	Unstandardized *B* (SE)	Standardized *β*	95% CI	*p-*value	*R^2^*	*F*
Intercept	59.52	31.54	−4.04, 123.1	0.07	0.24	*F* (6,44) =2.339 *p* = 0.048
HIV RNA load	−5.767	4.30	−14.43, 2.90	0.19		
CD4^+^ T cell count	−0.098	0.065	−0.23, 0.03	0.14		
CD8^+^ T cell count	0.028	0.022	−0.017, 0.072	0.22		
CD4^+^/CD8^+^ ratio	10.62	25.94	−41.65, 62.89	0.68		
WBC count	−3.81	3.705	−11.27, 3.67	0.31		
CD4^+^ T-cell-low patient group (<200/mL, whether or not)	56.08	28.40	−1.160, 113.3	0.05		

## 4 Discussion

This study has three points that differ from those of previous studies. First, 69 T-cell epitopes presented by 12 prevalent HLA-A allotypes were validated from three major HIV-1 proteins, with population coverages of 91, 83, 80, 70, and 63% in Northeast Asians, Southeast Asians, Europeans, South Americans, and North Americans, respectively. Previous studies have focused mostly on the prevalent HLA-A, B, and C allotypes in European, American, and African populations (Bugembe et al., 2020; Kaseke et al., 2021; Kinloch et al., 2019). Few studies have reported T-cell epitopes restricted by prevalent HLA allotypes in Asian and Chinese populations, especially for the dominant HIV-1 CRF01_AE and CRF07_BC genotypes in Asian populations (Hemelaar et al., 2019). The majority of the 69 validated peptides are relatively higher conservation in 14 HIV-1 subtypes, and 10 out of 26 reported peptides had been included in the relevance of existing vaccines. This study presents a broad-spectrum CD8^+^ T-cell epitope library for the design of T-cell epitope-based and preventative or therapeutic HIV vaccines tailored to Northeast Asian and global populations.

Second, a T-cell epitope can be presented by several HLA allotypes with different binding affinities, known as HLA cross-restriction. However, owing to the lack of standard methods, defining HLA cross-restriction is difficult. Therefore, majority of the previously reported HIV specific-T-cell epitopes have not been detailed in terms of their HLA restrictions, especially the cross-restriction of multiple HLA allotypes. Some epitopes, such as the HLA-A2 (Leitman et al., 2017), A33, and A30-restricted HIV epitopes, were only speculated on the basis of *in silico* prediction or the patient’s HLA serotype or genotype. However, this study not only verified the immunogenicity of 69 T-cell epitopes through functional experiments but also further identified their HLA-A cross-restriction using various methods, particularly the peptide competitive binding assay in which 12 cell lines expressing the indicated HLA-A allotype were used. The affinity of peptides for binding to HLA class І molecules is a key determinant of the epitopes of vaccine component (Sirskyj et al., 2011; Yang et al., 2021). Colleton et al. consistently reported that potential HIV-derived epitopes with high binding affinity to their HLA class I molecules were often linked to better immune responses, increased CD4^+^ T-cell counts, and slower disease progression (Colleton et al., 2009). In the present study, the binding affinity of 112 epitope candidates—comprised of 45 high-affinity epitopes and 31 intermediate-affinity epitopes—was validated using a peptide competitive binding assay. These data will be important for designing therapeutic or prophylactic HIV vaccines for Northeast Asian populations.

Third, this study provides an initial universal detection method using a functionally validated epitope peptide library to evaluate HIV-1-specific CD8^+^ T-cell function in Northeast Asian patients. Currently, the most widely used detection method involves coculturing of T-cell epitope peptides with patient PBMCs, followed by enzyme-linked immunospot assays (ELISPOT) or intracellular cytokine staining (ICS). However, most researchers have used overlapping peptide (OLP) libraries of HIV proteins for detection (Addo et al., 2003; Patel et al., 2016). Unfortunately, the overlapping peptides are not true epitope peptides verified by cell functional experiments, and recent studies have confirmed that majority of them are false epitopes (Cheng et al., 2021; Le Bert et al., 2020; Tarke et al., 2021). In addition, POL, GP160, and GAG contain approximately 334, 284, and 166 OLPs for CD8^+^ T-cell epitopes, respectively, when overlapping 6 amino acids in each 9-mer peptide, which makes the detection experiment expensive and laborious. In this study, a peptide library containing 141 functionally validated epitope peptides was established, and each epitope was able to cross-react with several dominant HLA-A molecules, covering the HLA polymorphisms of the Northeast Asian populations. The quantitative detection of activated HIV-1 specific CD8^+^ T cells in PBMCs was performed using peptide-PBMC *ex vivo* coculture and an ICS for individuals with HIV-1. This detection method can be used to test random Northeast Asian patients and provide results closer to real-world HIV specific CD8^+^ T-cell immunity.

In this study, HIV-1-specific CD8^+^ T-cell counts were significantly correlated with opportunistic infections and did not show a clear correlation with the HIV viral load, CD4^+^ T-cell count, CD4^+^ T-cell/CD8^+^ T-cells ratio, or total WBC count. This finding suggests that the count of HIV-1-specific CD8^+^ T cells significantly decreases as HIV infection progresses to opportunistic infections. Notably, whether HIV-specific T cells are correlated with the HIV RNA load remains controversial. Consistent with this study, Addo et al. screened HIV-1-specific T-cell responses in 57 people with HIV-1 using 504 OLPs spanning all HIV-1 proteins *via* the ELISPOT assay and reported no correlation between HIV-specific T-cell responses and the plasma HIV-1 viral load (Addo et al., 2003). Nevertheless, GAG-specific T-cell responses were reported to be negatively correlated with the plasma HIV RNA load, and Env-specific and accessory/regulatory protein–specific responses were associated with increased viremia (Addo et al., 2003). In addition, 5 of the 52 people with HIV-1 included in this study had no apparent response to the simulation of HIV peptide pools in the peptide-PBMC cocultures. This finding may imply that many people with HIV-1 are chronically infected and are receiving antiretroviral therapy and that HIV-specific CD8^+^ T-cell responses are weakened or exhausted (Al-Kolla et al., 2022). To obtain more convincing and comprehensive evidence, we will further expand the patient cohort to decipher the correlation between the HIV-specific CD8^+^ T-cell response and clinical and laboratory features.

However, this study has some limitations. On the one hand, during the *ex vivo* coculturing of patient PBMCs with epitope peptides to validate the immunogenicity of these peptides, the role of HLA-B and HLA-C molecules in presenting the epitope peptides was overlooked. This oversight potentially undermines the comprehensiveness of our understanding of the immunogenicity assessment process. On the other hand, the existing epitope peptide library, which consists solely of HLA-A-restricted epitope peptides, may be insufficient to fully decipher the overall response of CD8+ T cells to HIV infection. We intend to conduct a more in-depth screening of epitopes presented by prevalent HLA-B and C allotypes to address this limitation. By doing so, we aim to enrich the HIV peptide library and provide a more comprehensive representation of the target T cell clones. Another limitation of the study is that there are CD8 T-cells recognizing these peptides; they are not able to assign any anti-HIV functionality to any of these peptides. We will investigate which peptides play an important role in the contribution of protective epitope-specific T cells to protection against HIV-1 infection for functional vaccine design.

In summary, despite limitations in methods and design, this study validated 69 broad-spectrum CD8^+^ T-cell epitopes from the principal HIV-1 proteins through cell functional experiments, thus providing fundamental data for the design and development of T cell-directed HIV vaccines tailored for Northeast Asian populations. In addition, this study established a universal detection method to clinically evaluate the HIV-1-specific CD8^+^ T-cell response and revealed the associations of HIV-specific CD8^+^ T-cell reactivity with CD4^+^ T cells and HIV progression.

## Data Availability

The datasets presented in this study can be found in online repositories. The names of the repository/repositories and accession number(s) can be found in the article/Supplementary material.

## Footnotes

^1^ https://www.HIV.lanl.gov/content/immunology/

^2^ http://www.allelefrequencies.net

^3^ http://hiv.lanl.gov/
