# Supplementary material for: Large-scale screening of HIV-1 T-cell epitopes restricted by 12 prevalent HLA-A allotypes in Northeast Asia and universal detection of HIV-1-specific CD8+ T cells
Source: Front Microbiol. 2025 Feb 11;16:1529721. doi: 10.3389/fmicb.2025.1529721 (PMC11850406; doi:10.3389/fmicb.2025.1529721)
Supplement: Supplementary file 1 [file Data_Sheet_1.PDF]

Large-scale screening of HIV-1 T-cell epitopes restricted by 12 prevalent HLA-A allotypes in Northeast Asia and the universal detection of HIV-1 specific $CD8^+$ T cells

Yan Ding[1]*, Jialai Yan[2], Ling Huang[1], Jinhong Yu[1], Yandan Wu[3], Chuanlai Shen[3], Anning Fang[4]*

Table S1 *In silico* predicted 112 HIV $CD8^+$ T-cell epitope candidates were synthesized for further validation.
Table S2 Characteristics of 46 HIV infected patients whose PBMCs displayed positive $CD8^+$ T cell responses in peptide-PBMCs cocultures.

Figure S1: The flow cytometric dot plots of IFN-γ intracellular staining for each validated epitope and its negative control well in each peptide-PBMCs coculture. The PBMCs were stimulated with indicated epitope candidates overnight ex vivo and followed by IFN-γ intracellular staining. The frequencies of IFN-γ^+ cells in $CD3^+$/$CD8^+$ populations were analyzed by flowcytometry. Totally, 112 epitope peptides were tested using the PBMCs from 96 patients with matching HLA-A alleles, and 46 epitopes were defined as immunogenic epitopes.

Table S1 *In silico* predicted 112 HIV CD8$^+$ T-cell epitope candidates were synthesized for further validation

Epitope name	Protein	HLA-A allotype	start	end	Epitope sequence	IEDB ANN	IEDB SMM	SYFPEITHI	EPIJEN	Virus genotype
						IC50<500nM		Score>20	IC50 <500nM	
P1	GP160	A0201	816	825	SLVDTIAIAV	21.84	28.61	24	8.66	BC
P2	GP160	A0201	175	183	ALFYKLDIV	35.87	44.68	27	9.68	AE
P3	GP160	A0201	268	276	SLAEGEIII	43.99	96.19	26	9.20	BC
P4	GP160	A0201	102	110	QMQEDVISL	45.94	75.18	26	9.50	AE/BC
P5	GP160	A0201	691	699	IIFAVLSIV	65.79	67.01	25	8.77	AE/BC
P6	GP160	A0201	195	203	RLINCNTSV	13.74	22.19	24	9.30	AE
P7	GAG	A0201	29	37	YMLKHLVWA	5.23	12.22	22	9.92	BC
P8	GAG	A0201	67	75	ALQTGTEEL	150.25	202.82	28	10.01	BC
P9	GAG	A0201	275	283	RMYSPVSIL	173.66	191.92	24	9.19	AE
P10	GAG	A0201	77	85	SLFNTVATL	39.13	42.47	31	9.39	AE/BC
P11	POL	A0201	587	595	YQLEKDPIV	19.70	51.54	18	9.46	AE/BC
P12	POL	A0201	448	456	ALTDIVPLT	41.49	162.97	21		AE/BC
P13	POL	A0201	65	74	TLWQRPLVTV	53.78	50.52	29		AE
P14	POL	A0201	400	409	QLPEKDSWTV	61.80	128.97	24		BC
P15	POL	A0201	336	344	YQYMDDLYV	7.42	28.32	14	8.51	BC
P16	POL	A0201	287	295	YTAFTIPSI	36.57	186.26	22	8.94	AE
P17	POL	A0201	961	969	LLWKGEGAV	71.44	62.53	26	9.75	AE/BC
P18	POL	A0201	645	653	ALQDSGSEV	78.88	90.60	25	9.01	AE/BC
P19	GP160	A1101	285	293	KTIIVHLNK	7.89	14.17	25	8.43	AE
P20	GP160	A1101	669	677	ITNWLWYIK	8.26	28.33	20	8.41	AE/BC
P21	GP160	A1101	202	210	SVIKQACPK	13.57	26.69	25	8.48	AE
P22	GP160	A1101	395	403	GTYTPNGTK	27.50	37.78	24	8.71	BC
P23	GP160	A1101	221	230	CTPAGYAILK	38.54	29.71	28	7.55	AE/BC

P24	GP160	A1101	608	616	SSWSNKSQK	43.40	75.04	24	8.12	BC
P25	GP160	A1101	247	255	SVQCTHGIK	63.67	65.06	23	8.50	AE
P26	GP160	A1101	88	96	VTENFNMWK	21.43	40.21	21	8.37	AE/BC
P27	GP160	A1101	36	45	TVYYGVPVWK	29.72	19.18	25	6.01	BC
P28	GP160	A1101	49	58	TTLFCASDAK	30.76	21.13	21	7.80	AE/BC
P29	GAG	A1101	477	485	ELYPLTSLK	80.74	118.93	19	7.83	BC
P30	GAG	A1101	6	15	SILRGGKLDK	109.64	72.09	24	7.08	BC
P31	GAG	A1101	473	481	TSLPKQEQK	127.40	95.57	22	7.62	AE
P32	GAG	A1101	384	392	IVKCFNCGK	130.62	127.15	21	8.09	BC
P33	GAG	A1101	281	290	SILDIRQGPK	34.18	89.09	22	7.30	AE
P34	GAG	A1101	83	91	ATLWCVHQR	68.54	162.67	23	7.81	AE
P35	POL	A1101	395	404	TVQPIQLPEK	16.15	68.84	22	7.63	BC
P36	POL	A1101	536	545	ATESIVIWGK	29.45	41.96	25	6.11	AE
P37	POL	A1101	838	847	GSNFTSAAVK	50.18	64.54	25	8.16	AE
P38	POL	A1101	753	761	ASCDQCQLK	107.58	61.42	27	8.20	BC
P39	POL	A1101	513	521	RTAHTNDVK	94.96	75.91	22	8.18	BC
P40	POL	A1101	318	326	AIFQSSMTK	8.59	13.19	20	8.77	AE/BC
P41	POL	A1101	899	907	AVFIHNFKR	25.33	48.90	22	8.21	AE/BC
P42	POL	A1101	277	286	SVPLDESFRK	27.62	21.72	23	7.84	AE
P43	POL	A1101	540	548	IVIWGKTPK	32.42	57.45	21	8.07	AE/BC
P44	POL	A1101	576	584	FVNTPPLVK	39.93	64.91	27	8.42	AE/BC
P45	POL	A1101	675	683	QIIEQLIKK	52.92	96.01	21	8.25	BC
P46	GP160	A2402	385	393	FYCNTSGLF	70.68	138.56	22	1.85	BC
P47	GP160	A2402	631	639	NYTNQIYEI	335.03	359.44	21	1.724	AE
P48	GP160	A2402	676	684	WYIKIFIII	130.30	127.54	25	1.74	BC
P49	GP160	A2402	383	391	FYCNTTKLF	78.13	189.51	22	1.9	AE
P50	GP160	A2402	581	589	RYLKDQQLL	45.62	206.84	25	1.83	BC

P51	GP160	A2402	674	682	WYIKIFIMI	109.76	128.42	25	1.746	AE
P52	GAG	A2402	273	282	MYSPTSILDI	99.54	505.50	22		BC
P53	GAG	A2402	276	285	MYSPVSILDI	108.80	531.77	22		AE
P54	GAG	A2402	28	36	KYRLKHLVW	127.78	233.15	12	1.45	AE
P55	GAG	A2402	28	36	HYMLKHLVW	28.12	102.48	12	1.56	BC
P56	GAG	A2402	258	267	IYKRWIILGL	255.22	739.13	20		AE/BC
P57	POL	A2402	561	570	WWMEYWQATW	20.10	69.30	2		AE
P58	POL	A2402	498	506	TYQIYQEPF	44.07	106.57	22	1.72	AE/BC
P59	POL	A2402	734	742	RYHSNWRTM	63.28	150.19	9	1.43	AE
P60	POL	A2402	565	574	YWQATWIPEW	74.57	477.22	1		AE/BC
P61	POL	A2402	48	56	RQGTISFNF	76.56	278.37	12		BC
P62	POL	A2402	537	546	IWGKTPKFRL	153.72	598.03	12		AE/BC
P63	POL	A2402	215	223	PYNTPVFAI	393.72	156.18	22	1.56	AE
P64	POL	A2402	891	900	VQMAVFIHNF	130.68	344.13	13		AE/BC
P65	POL	A2402	286	295	KYTAFTIPSI	109.35	290.22	22		AE
P66	GAG	A0101	293	301	FRDYVDRFY	2446.84	577.50	25	7.118	
P67	GAG	A0101	120	129	EADGKVSQNY	2652.82	1741.0	25		
P68	GP160	A0101	187	195	YSENSSEYY	2.73	49.49	28	9.071	BC
P69	POL	A0101	651	660	VTDSQYALGI	852.63	1495.55	19		AE/BC
P70	GP160	A0203	22	30	GMLMISSAV	10.23	15.20		8.586	BC
P71	GAG	A0206	272	280	KIVRMYSPV	12.88	50.18		8.125	AE
P72	GAG	A0206	265	273	WIILGLNKI	57.03	210.16		7.937	AE/BC
P73	GP160	A0206	418	426	QIINMWQEV	11.80	7.93			BC
P74	POL	A0206	791	799	KVILVAVHV	15.29	17.72		8.124	AE
P75	POL	A0206	786	794	KIILVAVHV	25.57	21.65		8.768	BC
P76	GAG	A0207	293	301	YVDRFFKTL	0.91				BC
P77	GP160	A0207	817	825	LVDTIAIAV	0.81				BC

P78	GP160	A0207	580	589	YLKDQKFLGL	0.64				AE
P79	GP160	A0207	120	128	KLTPLCVTL	0.55				AE/BC
P80	POL	A0207	527	535	QLTEVVQKV	0.59				AE
P81	POL	A0207	268	276	VLDVGDAYF	0.57				AE
P82	POL	A0207	798	806	YMEAEVIPA	4154.75				BC
P83	GAG	A0301	18	26	KIRLRPGGK	28.05	61.35	29	9.57	BC
P84	GP160	A0301	801	809	LLYWGQELK	11.67	31.46	26	7.796	AE
P85	POL	A0301	710	718	KLVSSGIRK	27.32	37.31	26	9.223	AE/BC
P86	GAG	A1102	119	127	AAGTGSSSK	0.39	307.98			AE
P87	GP160	A1102	342	350	KVLKQVTEK	0.94	43.19			AE
P88	POL	A1102	22	30	RANSPTSRK	0.88	67.76			AE
P89	GAG	A3001	20	28	RLRPGGRKK	5.68	15.24			AE
P90	GAG	A3001	37	45	ASRELERFA	26.00	44.49			AE/BC
P91	GP160	A3001	701	709	RVRQGYSPL	4.08	15.63			AE/BC
P92	GP160	A3001	766	774	RLRGFILVA	6.58	28.90			BC
P93	POL	A3001	978	986	KVVPRRKAK	17.91	39.26			AE/BC
P94	POL	A3001	690	698	KVYLSWVPA	7.47	25.35			AE
P95	POL	A3001	823	831	KLAGRWPVK	23.61	76.73			AE/BC
P96	GAG	A3101	387	395	RIKCFNCGR	3.35	14.37		6.791	AE
P97	GAG	A3101	375	383	RSNFKGSKR	6.15	8.66		7.571	BC
P98	GP160	A3101	494	502	RAKRRVVER	5.70	5.38		7.03	AE
P99	GP160	A3101	3	11	VTGIRKNYR	14.21	17.93		8.11	BC
P100	POL	A3101	12	20	KAREFSSER	9.46	30.44			BC
P101	POL	A3101	508	516	NLKTGKYAR	37.98	52.90		7.537	AE
P102	POL	A3101	324	332	MTKILEPFR	12.17	16.24		7.579	AE/BC
P103	GAG	A3101	139	147	MVHQPISPR	17.71	26.82		7.393	BC
P104	GAG	A3303	14	22	DAWEKIRLR	0.85	80.90			AE
P105	GAG	A3303	366	375	QTNSAILMQR	0.64	45.14			BC

P106	GP160	A3303	428	436	MYAPPISGR	0.92	9.10	AE
P107	GP160	A3303	442	450	NITGILLTR	0.83	72.88	AE
P108	GP160	A3303	168	176	QTVYALFYR	0.74	11.55	BC
P109	POL	A3303	893	902	MAVFIHNFKR	5.77	5.77	BC
P110	POL	A3303	599	608	TFYVDGAASR	0.68	27.28	AE
P111	GAG	A3303	142	150	MAHQPLSPR	0.85	10.07	AE
P112	POL	A3303	940	948	IQNFRVYYR	0.6	16.23	AE/BC

Note: AE, CRF01_AE; BC, CRF07_BC

Table S2 Characteristics of 46 HIV infected patients whose PBMCs displayed positive $CD8^+$ T cell responses in peptide-PBMCs cocultures

Patient ID	Gender	Age (years)	CD4 ($\times 10^6$/L)	CD8 ($\times 10^6$/L)	Viral load (copies/mL)	WBC ($\times 10^9$/L)	CRP (mg/L)	HLA-A genotype	Epitopes inducing positive $CD8^+$ T cell response
HIV1	M	36	129	561	<20	4.79	3.55	A2402	P48
HIV2	M	44	/	/	<20	4.94	/	A0201/2402	P69/70/71/
HIV3	M	36	525	879	<20	5.3	2.96	A0201/1101	P10
HIV4	M	29	786	620	<20	6.89	0.55	A3001/3002	P19/20
HIV5	M	34	335	888	<20	6.98	4.53	A0201/2402	P23
HIV6	M	60	274	1208	27.7	5.53	75.72	A1101/3101	P25/26/27
HIV7	M	57	65	498	1.60×10^6	2.87	4.11	A0207/2601	P50/51/52/53
HIV8	M	62	635	368	<20	4.93N	1.22	A0206/2601	P5/P6
HIV9	M	59	321	524	<20	3.28	48.7	A0201/6601	P15
HIV10	F	11	7	755	5.41×10^6	2.64	0.5	A0207/2301	P29/30/31/32
HIV11	F	43	103	874	2.13×10^2	3.55	3.73	A1101/2402	P29/30/31/32
HIV12	M	57	255	322	<20	4.16	0.84	A1101/3101	P36
HIV13	M	28	148	1158	<20	4.49	1.24	A0201/1101	P71
HIV14	M	55	301	324	<20	10.13	65.92	A1101/3101	P32/33
HIV15	M	43	128	358	<20	4.33	0.53	A1101/3101	P33/34/35/36
HIV16	F	51	514	588	<20	5.3	0.3	A0201/3301	P78/79
HIV17	M	30	1	395	4.02×10^5	2.19	5.79	A2401/3101	P83/84/85/87
HIV18	M	25	557	640	/	5.8	0.64	A0201/2401	P55
HIV19	F	45	552	1336	/	6.63	12.93	A3301	P58/60
HIV20	M	48	375	339	<20	5.98	0.57	A0201/2402	P61
HIV21	M	57	231	264	<20	4.72	74.23	A3301/3101	P90
HIV22	M	60	145	810	27.8	4.49	0.63	A0201/3001	P79/80
HIV23	M	33	283	1017	<20	9.63	47.29	A2402/1101	P109
HIV24	M	74	184	422	<20	4.25	79.99	A0201/1101	P62
HIV25	M	60	412	1409	<20	7.77	14.77	A0201/2402	P81/63

HIV26	M	56	254	650	2.27×10^2	4.66	6.13	A1101/3303	P109
HIV27	M	40	225	1168	<20	6.89	19.25	A1101/3301	P91/93/111
HIV28	M	32	282	1232	3.21×10^5	9.05	31.93	A1101/3301	P93/66
HIV29	M	54	366	647	<20	8.18	1.3	A3001/3101	P74
HIV30	M	69	328	808	<20	7.73	2.63	A0201/3001	P91/94/95
HIV31	M	77	/	/	/	5.58	1.17	A0207/1101	P92/95
HIV32	M	66	82	1002	73.6	5.44	7.45	A0201/1101	P49/54
HIV33	M	47	19	224	<20	0.65	130.3	A1101/3101	P65/45
HIV34	M	38	884	1434	39.8	9.57	3.99	A0301/3303	P55/65/61/56
HIV35	M	56	291	644	<20	4.27	/	A0201/2401	P5/6/7
HIV36	M	54	106	288	62.2	6.65	9.34	A0201/3301	P44
HIV37	M	68	142	379	/	6.56	26.14	A1101/3101	P102/103
HIV38	M	50	18	358	61.2	7.46	21.34	A0201/3101	P100/103
HIV39	F	52	481	625	<20	4.12	2.26	A0201/2402	P104/106
HIV40	M	50	762	840	21.7	7.24	2.28	A0201/0207	P104/105/9
HIV41	M	52	371	412	<20	4.01	7.26	A0201/2402	P104/105/106/9
HIV42	F	29	65	342	<20	4.74	1.62	A0201/0301	P9
HIV43	M	27	14	530	1.60×10^2	1.69	186.6	A3001	P66/68/69
HIV44	M	58	1020	831	<20	6.74	1.03	A2402/0301	P10
HIV45	M	46	279	654	<20	2.91	/	A0201/0207	P11/102/12
HIV46	F	/	/	/	/	/	/	A2402/0201	P68

Note: **F**: Female，**M**: Male， **n.d**: not detection，VL: viral load CRP: C-reaction protein

A:

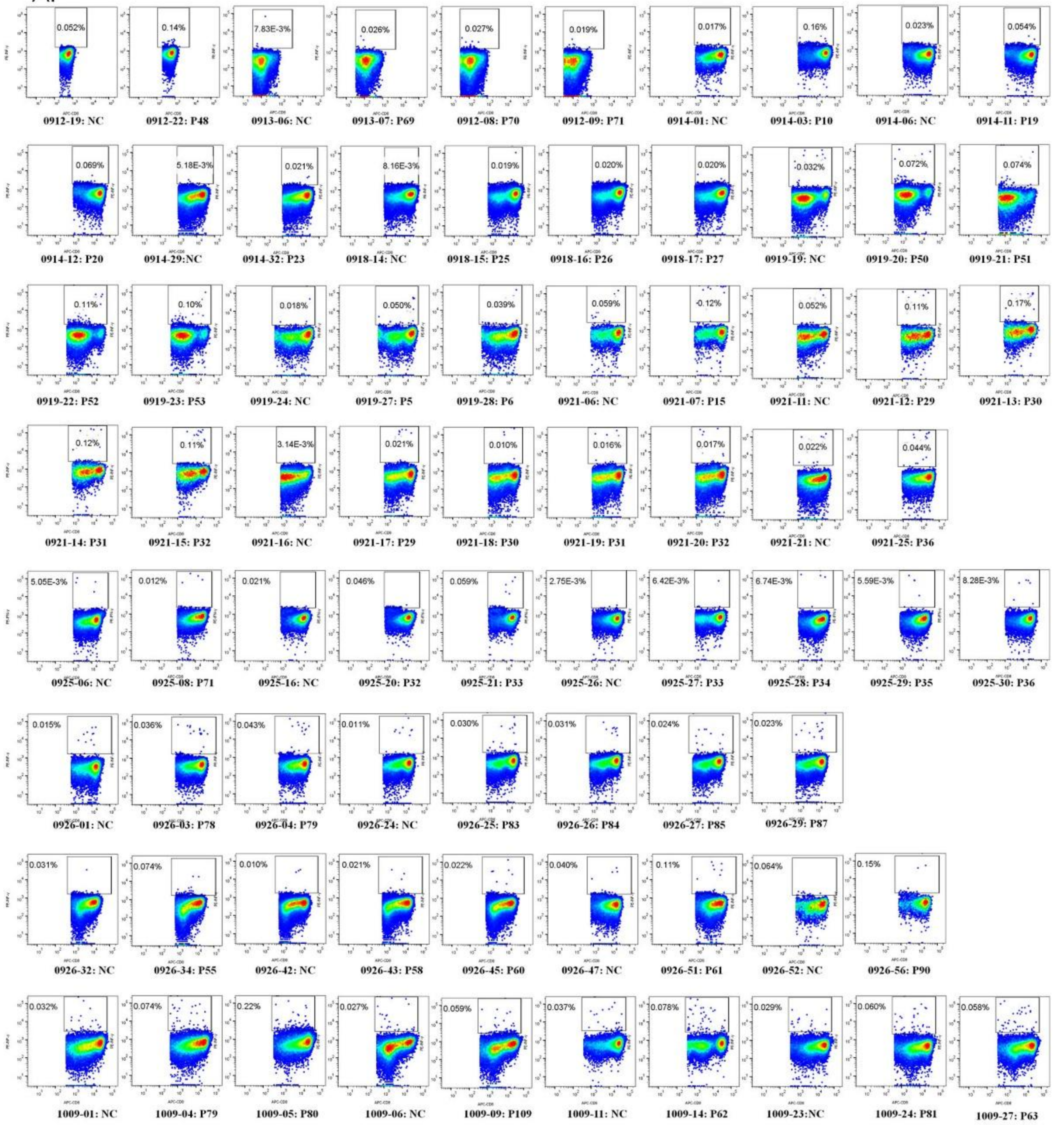
0.052%
0.14%
7.83E-3%
0.026%
0.027%
0.019%
0.017%
0.16%
0.023%
0.054%
0912-19: NC
0912-22: P48
0913-06: NC
0913-07: P69
0912-08: P70
0912-09: P71
0914-01: NC
0914-03: P10
0914-06: NC
0914-11: P19
0.069%
5.18E-3%
0.021%
8.16E-3%
0.019%
0.020%
0.020%
0.032%
0.072%
0.074%
0914-12: P20
0914-29:NC
0914-32: P23
0918-14: NC
0918-15: P25
0918-16: P26
0918-17: P27
0919-19: NC
0919-20: P50
0919-21: P51
0.11%
0.10%
0.018%
0.050%
0.039%
0.059%
0.12%
0.052%
0.11%
0.17%
0919-22: P52
0919-23: P53
0919-24: NC
0919-27: P5
0919-28: P6
0921-06: NC
0921-07: P15
0921-11: NC
0921-12: P29
0921-13: P30
0.12%
0.11%
3.14E-3%
0.021%
0.010%
0.016%
0.017%
0.022%
0.044%
0921-14: P31
0921-15: P32
0921-16: NC
0921-17: P29
0921-18: P30
0921-19: P31
0921-20: P32
0921-21: NC
0921-25: P36
5.05E-3%
0.012%
0.021%
0.046%
0.059%
2.75E-3%
6.42E-3%
6.74E-3%
5.59E-3%
8.28E-3%
0925-06: NC
0925-08: P71
0925-16: NC
0925-20: P32
0925-21: P33
0925-26: NC
0925-27: P33
0925-28: P34
0925-29: P35
0925-30: P36
0.015%
0.036%
0.043%
0.011%
0.030%
0.031%
0.024%
0.023%
0926-01: NC
0926-03: P78
0926-04: P79
0926-24: NC
0926-25: P83
0926-26: P84
0926-27: P85
0926-29: P87
0.031%
0.074%
0.010%
0.021%
0.022%
0.040%
0.11%
0.064%
0.15%
0926-32: NC
0926-34: P55
0926-42: NC
0926-43: P58
0926-45: P60
0926-47: NC
0926-51: P61
0926-52: NC
0926-56: P90
0.032%
0.074%
0.22%
0.027%
0.059%
0.037%
0.078%
0.029%
0.060%
0.058%
1009-01: NC
1009-04: P79
1009-05: P80
1009-06: NC
1009-09: P109
1009-11: NC
1009-14: P62
1009-23:NC
1009-24: P81
1009-27: P63

B:

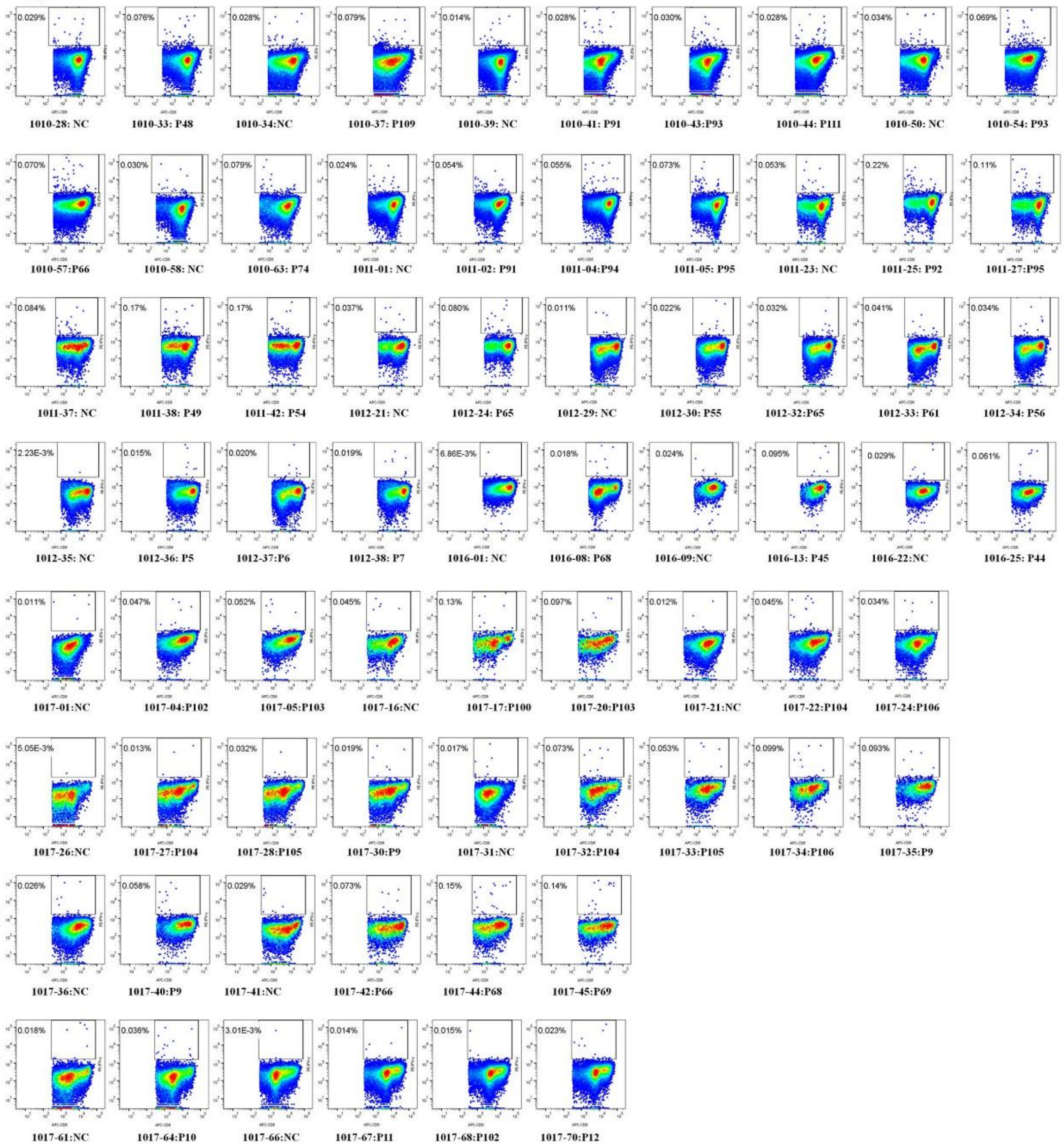

Figure S1: The flow cytometric dot plots of IFN-γ intracellular staining for each validated epitope and its negative control well in each peptide-PBMCs coculture. The PBMCs from 96 HIV-1 infected patients were co-cultured 20 h with the with the corresponding epitope candidate peptides which can presented by the patient's HLA-A allotypes as virtually predicted. Of which, 46 PBMCs displayed positive T cell responses in the peptide-PBMCs cocultures. The frequencies of IFN-γ+ cells in the CD3+/CD8+ population of each validated epitope and its negative control well in each PBMCs sample were presented. NC, negative control well without peptide. Totally, 112 epitope dandidate peptides were tested using the PBMCs from 96 patients with

matching HLA-A alleles.
